# Phenotypic characterization of patient dengue virus isolates in BALB/c mice differentiates dengue fever and dengue hemorrhagic fever from dengue shock syndrome

**DOI:** 10.1186/1743-422X-8-398

**Published:** 2011-08-11

**Authors:** Anne Tuiskunen, Maria Wahlström, Jakob Bergström, Philippe Buchy, Isabelle Leparc-Goffart, Åke Lundkvist

**Affiliations:** 1 Department of Microbiology, Tumor and Cell Biology, Karolinska Institutet, Stockholm, Sweden; 2 Swedish Institute for Communicable Disease Control, Solna, Sweden; 3 Virology Department, French Army Forces Biomedical Institute (IRBA), Marseille, France; 4 Virology Unit, Institut Pasteur in Cambodia, Phnom Penh, Cambodia

**Keywords:** dengue virus, mouse model, tropism, clinical isolate, cytokines, dengue hemorrhagic fever, flavivirus

## Abstract

**Background:**

Dengue virus (DENV) infection is the most common arthropod-borne viral disease in man and there are approximately 100 million infections annually. Despite the global burden of DENV infections many important questions regarding DENV pathogenesis remain unaddressed due to the lack of appropriate animal models of infection and disease. A major problem is the fact that no non-human species naturally develop disease similar to human dengue fever (DF) or dengue hemorrhagic fever (DHF) and dengue shock syndrome (DSS). Apart from other risk factors for severe dengue such as host genetics and secondary infection with a heterologous DENV, virus virulence is a risk factor that is not well characterized.

**Results:**

Three clinical DENV-1 isolates from Cambodian patients experiencing the various forms of dengue disease (DF, DHF, and DSS) were inoculated in BALB/c mice at three different concentrations. The DENV-1 isolates had different organ and cell tropism and replication kinetics. The DENV-1 isolate from a DSS patient infected the largest number of mice and was primarily neurotropic. In contrast, the DENV-1 isolates from milder clinical dengue cases infected predominantly lungs and liver, and to a lesser extent brain. In addition, infection with the DENV isolate derived from a DSS patient persisted for more than two weeks in a majority of mice compared to the other DENV-1 isolates that peaked during the first week.

**Conclusions:**

These results confirm the *in vitro *findings of the same DENV-1 isolates, that showed that the isolate derived from a DSS patient can be distinguished based on phenotypic characteristics that differ from the isolates derived from a DF and DHF case [1]. We observed in this study that the DSS virus isolate persist longer *in vivo *with extensive neuroinvasion in contrast to the other DENV-1 isolates originating in milder human cases. Genomic characterization of the three clinical isolates identified six amino acid substitutions unique for the DSS isolates that were located both in structural genes (M and E) and in non-structural genes (NS1, NS3, and NS5). The characterization of these clinically distinct DENV-1 isolates highlight that DENVs within the same genotype may have different *in vivo *phenotypes.

**Highlights:**

• Clinical DENV-1 isolates have different organ tropism in BALB/c mice.

• The isolate from a DSS patient is primarily neurotropic compared to the other isolates.

• The DENV-1 isolates have different *in vivo *replication kinetics.

• The isolate from a DSS patient persists longer compared to the other isolates.

• These phenotypic differences confirm our earlier *in vitro *findings with the same DENV-1 isolates. Thus, DENVs within the same serotype and genotype may differ enough to affect clinical conditions *in vivo*.

## Background

The dengue viruses (DENV) belong to the genus flavivirus of the *Flaviviridae *family, and consist of four (1-4) antigenically related, but clearly distinct viruses (serotypes). The DENV particle is enveloped, and has a single-stranded positive-sense RNA genome of approximately 11 kb that resembles a messenger RNA with a cap on the 5' end but no poly(A) tail at the 3' end. The RNA genome encodes a 3411 long precursor polyprotein that contains three structural proteins (C, prM, and E), and seven nonstructural proteins (NS1, NS2A, NS2B, NS3, NS4A, NS4B, and NS5). The open reading frame is flanked by two nontranslated regions (5' and 3' NTR) of approximately 95-135 and 114-650 nucleotides, respectively, that have characteristic secondary structures that are required for efficient translation and replication [2,3].

The DENVs are endemic in tropical and subtropical areas and more than one hundred million people get infected annually. Infection can be either asymptomatic, or cause an acute febrile illness that is characterized by fever, headache, retro-orbital pain, arthralgia, and myalgia. This condition can progress into dengue haemorrhagic fever (DHF), with cardinal signs such as increased vascular permeability, thrombocytopenia, focal or generalized haemorrhages. DF may progress into the life-threatening state of dengue shock syndrome (DSS) [4]. In the recent WHO guidelines, for case management purposes, DHF and DSS cases are now grouped together as "severe dengue" (group C) [5].

Uncontrolled urbanization and globalization have resulted in the geographic spread of the DENV-transmitting mosquitoes *Aedes aegypti *and *A. albopictus*, co-circulation of different DENV serotypes, and increased frequency of dengue epidemics [6]. There has been a severe increase of DHF/DSS in many endemic regions, emphasizing the urgent need of an effective vaccine.

Despite the global burden of DENV infections many important questions regarding DENV pathogenesis remain unaddressed due to the lack of appropriate animal models of infection and disease. A major problem is the fact that no non-human species naturally develop disease similar to human DF or DHF/DSS. Epidemiological, clinical, and laboratory findings indicate that both genetic differences in the virus and the host immune response contribute to the occurrence and severity of disease [7].

In the search for better animal models for dengue, several approaches to investigate infection of DENV in mouse have been proposed. One of two major strategies have been to modulate the mouse to render it more susceptible to DENV infection, e.g. SCID mice transplanted with cultured, DENV-susceptible tumour cells [8-10]. The other major alternative has been to modify the DENV in order to make it more infection-competent in the murine host, e.g. mouse-neuroadapted DENV [11-14], or virus serially passaged in mouse [15,16]. The relevance of these models to human pathogenesis remains unknown.

The immunocompetent BALB/c mouse is susceptible to DENV infection and has been used extensively to study various aspects of DENV infection pathogenesis, despite the lack of clinical symptoms [17-20]. Mouse-adapted DENV strains increase the susceptibility to DENV infection and elicit clinical symptoms in BALB/c mice [11,21]. The relevance of these observations to infection with wild-type DENV strains, however, should be interpreted with caution due to the altered genotype and phenotype of the passaged virus.

We have previously reported that DENV-1 isolates only passaged once in C6/36 cells (passage 1), derived from patients presenting the clinically distinct forms of dengue (DF, DHF, and DSS), can be distinguished *in vitro *based on different replication kinetics in mammalian Vero cells and apoptosis in C6/36. The DSS-derived isolate (accession number: FJ639694) had a unique phenotype compared to the DF- and DHF-derived isolates (accession numbers: HQ624983 and HQ624984, respectively) and genomic comparison revealed six amino acid substitutions unique for the DSS isolate that were located in the structural viral M and E genes that constitute the extracellular mature virus particle, and in the NS1, NS3, and NS5 genes. The E protein mediates host cell receptor binding, viral entry, and is a major target for humoral immunity [22,23]. The role of NS1 in virus replication is not known, but is thought to be involved in facilitation of viral infection and in the pathogenesis of DENV infection [24,25]. The NS3 acts together with its cofactor NS2B as the viral serine protease needed for DENV precursor polyprotein-processing [3,26-28]. The NS5 protein has a dual enzymatic activity with an RNA dependent RNA polymerase activity in its C-terminal domain, and a methyltransferase activity at its N-terminal end. Hence, these six mutations could ultimately affect antibody response, host immune pathways, and virus replication.

The primary aim of the present study was to investigate whether there is a phenotype difference *in vivo *between the DENV-1 isolates similar to the *in vitro *findings, by studying difference in infectivity in BALB/c mice and to investigate the cell and organ tropism. The second aim was to determine if the inoculated virus concentration and the effects of the infection correlated, primarily by measuring the levels of inflammatory cytokines induced by the infection.

## Results

### DENV infection and Organ Tropism

All mice had detectable levels of DENV specific IgG antibodies at day 15 post-infection (p.i.), confirming the intravenous route to be appropriate choice in order to successfully infect all mice. In total 54.3% of the DENV-1 infected mice had detectable levels of viral RNA; 48.2% of the mice infected with the DF isolate; 55.6% of the DHF isolate infected mice; and 59.3% of the DSS isolate infected mice (table 1). All mice survived the infection without presenting any clinical signs but pathological lesions such as haemorrhage could be detected, most notably in brain, liver, lung tissue, and to some extent in spleen.

**Table 1:** Viral RNA was found in various organs depending on inoculated DENV-1 isolate (DF, DHF, or DSS), virus concentration in inoculum, and time-point p.i. In total 54.3% of the DENV-1 infected mice had detectable levels of viral RNA.

DENV isolate	DF									DHF									DSS								
Titer (PFU/mL)*	**1.5 × 10**^**4**^			**1.5 × 10**^**5**^			**1.5 × 10**^**6**^			**1.5 × 10**^**4**^			**1.5 × 10**^**5**^			**1.5 × 10**^**6**^			**1.5 × 10**^**4**^			**1.5 × 10**^**5**^			**1.5 × 10**^**6**^		

Day (p.i.)**	3	6	15	3	6	15	3	6	15	3	6	15	3	6	15	3	6	15	3	6	15	3	6	15	3	6	15

Heart	-	-	-	-	-	-	-	-	-	-	-	-	-	-	-	-	-	-	-	-	-	-	-	-	-	-	-

Lungs	-	-	-	2	3	-	2	3	1	-	-	-	-	2	-	2	2	1	-	-	-	-	3	1	1	1	1

Spleen	1	-	-	-	-	-	2	-	-	1	-	-	-	-	2	2	1	-	-	1	-	-	1	1	2	-	-

Brain	-	-	-	-	-	-	1	-	-	1	1	-	-	-	-	-	3	-	-	1	2	1	3	2	-	2	-

Liver	-	-	-	-	-	-	-	-	-	-	-	-	1	1	-	2	-	-	-	-	-	-	-	-	-	-	-

Kidneys	-	-	-	-	-	-	-	-	-	-	-	-	-	-	-	-	-	-	-	1	-	-	-	-	-	-	-

Infected mice	1	-	-	2	3	-	3	3	1	2	1	-	1	2	2	3	3	1	-	3	2	1	3	3	2	2	1

Infected mice per titer	1			5			7			3			5			7			4			7			5		

Infected mice per day p.i.	6			6			1			6			6			3			3			7			6		

Σ infected mice per virus isolate	13									15									16								

% infected mice per virus isolate	48.2%									55.6%									59.3%								

* Titer of inoculum was significant (*p = 0.012*).

** Time-point post-infection was significant (*p = 0.014*).

Haemorrhage in lungs was most pronounced in the DF-infected mice, whereas the DHF-infected mice experienced haemorrhage in lungs, liver and to some extent in brain. The DSS isolate seemed to have some neurotropism as the DSS-infected mice had extensive bleedings in the brain, whereas the peripheral organs showed only minor signs of haemorrhage. None of the NC mice exhibited any clinical signs or tissue pathology.

The three DENV-1 isolates showed different tropism; the two DENVs obtained from a DF and DHF case infected primarily lungs, spleen, and liver, whereas the DENV from a DSS patient infected lungs, spleen, and exhibited a strong tropism for brain tissue (*p = 0.001*). The results are summarized in table 1. The effects of infection in lung tissue showed a positive correlation to inoculated virus concentration and time-point, with a peak of infection at day 6 p.i. (*p = 0.003*) combined with the highest titer 1.5 × 10^6 ^PFU/mL in the inoclum (*p < 0.001*) (table 2).

**Table 2:** Risk of infection in lungs.

Inoculated virus (PFU/mL)	Risk of infection
1.5 × 10^4^	0
1.5 × 10^5^	0.41
1.5 × 10^6^	0.52
Days p.i.	Risk of infection
3	0.26
6	0.52
15	0.15

Both inoculated DENV-1 concentration and time-point were significant (*p < 0.001 *and *p = 0.003*, respectively). The risk for infection in the lungs with the DF isolate was highest on day 6 p.i. and showed a positive correlation to inoculated virus.

### Risk of Infection and Kinetics of Infection

**Figure 1:**
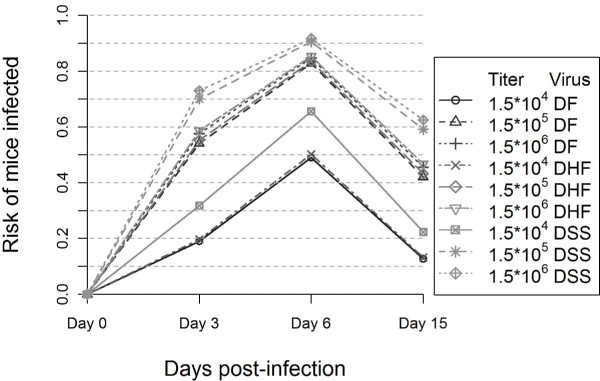
**Estimated risk of infection depending on virus concentration, day p.i. and virus isolate**.

The DENV-1 isolates exhibited different risks for infection that depended both on virus concentration in the inoculum (*p = 0.012*) and on time-point p.i. (*p = 0.014*) (Figure 1). The effects of inoculated dose of infectious virus showed a positive correlation to the total number of infected mice for each virus isolate, and infection peaked on day 6 p.i. for all three viruses.

**Figure 2:**
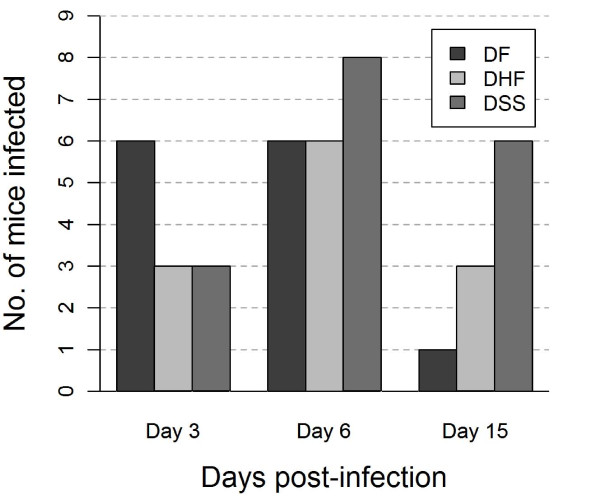
**Persistence of infection shown as the number of mice infected out of the total mice inoculated, independent of inoculated virus concentration**. Nine mice per DENV isolate and time-point in total were inoculated with the corresponding DENV isolates; DF, DHF, and DSS, respectively.

Kinetics of infection differed between the three DENV-1 isolates. Viral RNA in the DF and DHF inoculated mice was predominantly detected during the first week of infection, and declined during the second week. Six inoculated mice tested positive after inoculation with the DF, and DHF isolates, respectively, both on day 3 and 6 p.i. During the second week (day 15 p.i.), however, only 1 and 3 mice tested positive after inoculation with the DF- and DHF-viruses, respectively. The time-course of infection differed in the DSS- infected mice that showed a delayed response to inoculation compared to the DF- and DHF-infected mice. Day 3 p.i. had the lowest number of DSS-infected mice (3 positive mice) whereas at day 6 p.i. 7 mice were infected, and 6 during the second week of infection (day 15 p.i.). Thus, the number of mice infected with the DF and DHF isolates peaked during day 3 and 6 p.i. in contrast to the number of DSS-infected mice that peaked during day 6 and 15 p.i (Figure 2).

Overall, the DSS isolate replicated in a majority of infected mice and for a longer period of time compared to the DENVs obtained from milder cases (Figure 2).

### Cytokine secretion in serum

**Figure 3:**
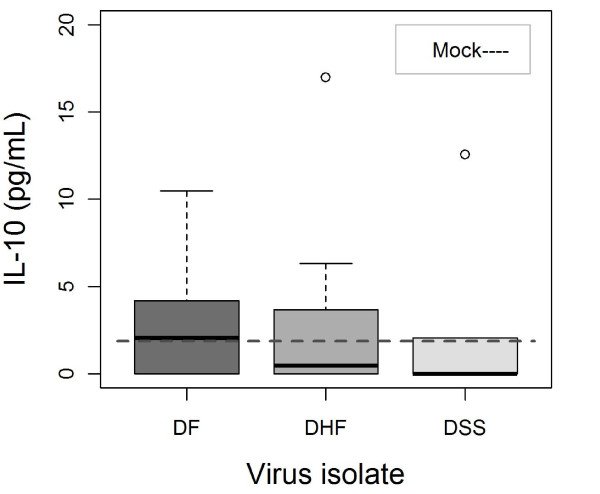
**Box plots showing median IL-10 (solid line) by DENV-1 isolates independent of inoculated virus concentration and days p.i**. The line in the middle of each box represents the median; the boxes consists of 1^st ^and 3^rd ^quartile, whiskers are 1.5*(3^rd ^quartile-1^st ^quartile). Outliers are marked with an open circle. The DF-inoculated mice had higher levels of IL-10 compared to the DHF- and DSS-inoculated mice (*p = 0.007*), with a peak at day 3 p.i (*p = 0.009*).

**Figure 4:**
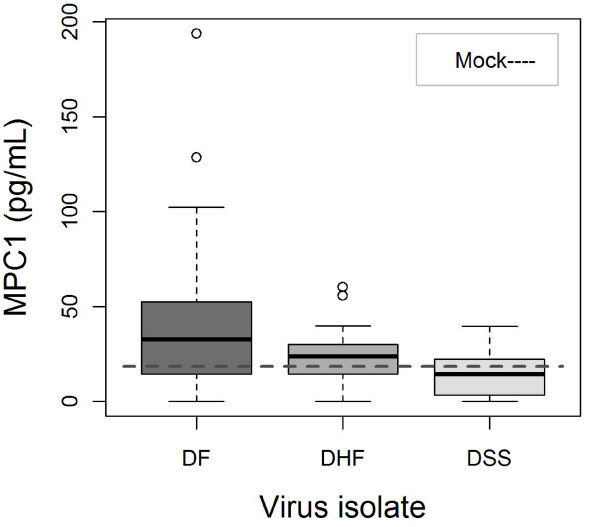
**Box plots showing median MCP-1 (solid line) by DENV-1 isolates independent of inoculated virus concentration and days p.i**. The line in the middle of each box represents the median; the boxes consists of 1^st ^and 3^rd ^quartile, whiskers are 1.5*(3^rd ^quartile-1^st ^quartile). Outliers are marked with an open circle. The DF-inoculated mice had the highest serum levels of secreted MCP-1 and showed the biggest difference compared to the DSS-inoculated mice (*p < 0.0001*), followed by DHF-inoculated mice (*p = 0.011*). MCP-1 levels peaked at day 3 p.i. and whereupon it decreased (*p = < 0.001*).

IFNγ levels peaked during the first week of infection and most notably on day 3 p.i. that showed the highest levels of IFNγ (*p = 0.007*). There was also a positive correlation to inoculated virus dose where the highest titer (1.5 × 10^6 ^PFU/mL) elicited the strongest IFNγ response (*p = 0.002*). A positive titer dependent correlation to IL-6 levels was observed in all three groups of DENV-1 inoculated mice (*p = 0.028*) (data not shown). There was a time and virus dependent difference regarding detected IL-10 levels. The DF-inoculated mice had higher levels of IL-10 compared to the DHF- and DSS-inoculated mice (*p = 0.007*), with a peak at day 3 p.i (*p = 0.009*) (Figure 3). The DF-inoculated mice also dominated in serum levels of secreted MCP-1 and showed the biggest difference compared to the DSS-inoculated mice (*p < 0.0001*), followed by DHF-inoculated mice (*p = 0.011*) (Figure 4). MCP-1 levels peaked at day 3 p.i. whereupon it decreased (*p = < 0.001*). Inoculated virus titer showed a positive correlation to measured serum levels of RANTES in all DENV-1 inoculated mice (*p = 0.0036*), and DF-inoculated mice dominated (*p = 0.048*). IL-13 and RANTES showed a dose-dependent response to inoculated virus. IL-1β, IL-2, and TNFα did not show any statistically significant relationships to any of the analysed parameters (table 3).

**Table 3:** Serum cytokine levels in mice inoculated with DENV-1 isolates from patients with DF, DHF, and DSS, respectively, and independent of inoculated virus concentration.

**Day p.i**.	Cytokine	DF	DHF	DSS	Mock
3	IFNγ	3.10	3.24	2.08	0.09

	IL-1β	3.15	0.00	0.00	0.00

	IL-2	1.46	2.00	1.05	0.00

	IL-6	1.48	1.65	2.69	3.22

	IL-10	4.20	3.13	0.00	0.00

	IL-13	88.83	73.12	80.23	98.84

	MCP-1	71.58	34.25	21.45	0.00

	RANTES	35.11	13.17	15.93	9.76

	TNFα	6.44	3.97	3.78	0.00

6	IFNγ	2.53	1.65	1.92	0.00

	IL-1β	3.15	0.00	0.00	0.00

	IL-2	1.46	1.44	2.21	2.21

	IL-6	0.36	0.77	3.10	2.69

	IL-10	2.05	0.00	0.00	0.00

	IL-13	70.84	62.33	51.19	121.60

	MCP-1	22.12	14.54	3.37	0.00

	RANTES	20.47	14.16	24.46	10.43

	TNFα	2.45	2.15	0.00	0.00

15	IFNγ	1.24	1.28	1.70	0.87

	IL-1β	0.00	0.00	0.00	0.00

	IL-2	2.20	1.19	5.61	0.00

	IL-6	0.18	1.88	1.65	0.03

	IL-10	1.03	0.00	0.00	0.00

	IL-13	67.20	91.70	84.96	75.57

	MCP-1	21.53	14.54	3.37	0.00

	RANTES	27.68	17.22	20.75	15.02

	TNFα	1.73	0.00	1.48	0.00

The cytokines were measured in pg/mL.

## Discussion

DENV infections are a major public health problem and constitute a real challenge due to the absence of vaccines and effective antiviral drugs. There are no non-human species that naturally develops disease following DENV inoculation into peripheral sites and that resemble the clinical symptoms observed in humans [29]. The mechanisms underlying the immune responses to DENV infection are still poorly defined, and the lack of suitable animal models has hampered investigation of host- and virus-specific mechanisms that control primary and sequential DENV infections.

In this study, we have compared three minimally passaged DENV-1 isolates from patients experiencing the various clinical forms of dengue disease (DF; DHF; and DSS, respectively) *in vivo *by infection in mouse. We show that the isolate from a DSS patient infected the largest number of BALB/c mice, with a different tropism compared to the DENV-1 isolates obtained from milder clinical cases during the same outbreak. This indicates that DENVs within the same serotype and genotype may differ enough to cause various clinical conditions *in vivo*. These results confirm a phenotypic distinction previously observed *in vitro *with the same DENV-1 isolates where the DSS isolate replicated less efficiently in mammalian Vero cells than the DF and DHF isolates, and elicited apoptosis in mosquito C6/36 cells [1].

The aim of the study was to compare wild-type features of DENV-1 isolates passaged only once *in vitro *and never before inoculated in mouse or cultured in mouse cells prior to direct intravenous inoculation into BALB/c mice. BALB/c mice are known to be susceptible for DENV infection although with discrete pathological features [11,30]. We found that the mice did not develop dengue disease, and did not present any symptoms of the infection. However, viral RNA was detected in various organs depending on the DENV isolate injected (table 1). We were unable to detect any systemic viremia and this suggests an absence or a very low level of virus replication in circulating peripheral blood mononuclear cells. Alternatively, the lack of detectable viraemia could indicate that the virus had already disseminated into peripheral sites, since tissue infection was measured from day 3 p.i.

In brief, viral RNA was detected by qRT-PCR in spleen, liver, lungs, kidneys, and brain. Viral RNA was found in the kidneys in only one mouse, which is in line with earlier studies [31]. Organ tropism clearly differed between the three clinical DENV-1 isolates and our findings suggest that the disease severity may correlate with high tissue viral burden, even if plasma viraemia was below the detection limit. The DENV isolate from a DSS case showed a strong preference for brain tissue, compared to the two isolates from milder dengue cases that primarily were found in other organs as lungs and liver. Neurotropism in mice has been previously suggested to be associated with DENV virulence when comparing DENV-3 isolated from Brazilian patients [32].

Breakdown of the blood-brain barrier in DENV infected mice have been described before, and were shown to be dose-dependent [33]. Genomic sequencing performed previously [1] identified a unique amino acid substitution L→F476 in the E protein of the DSS isolate compared to the DF and DHF isolates, that may explain the neurotropic character of the DSS-isolate. The NS1 gene of the DSS isolate harboured another unique amino acid substitution, K→R115 in comparison to the two other DENV-1 isolates. The soluble form of NS1 (sNS1) is a dominant target of humoral immunity and activates complement components in normal human serum, and is proposed to play a significant role in the pathogenesis of disease [25,34]. The NS1 glycoprotein is glycosylated at two sites, N130 and N270, and glycosylation of both residues has been suggested to be required for neurovirulence in mice [35]. It is remains to be elucidated whether the possible breakdown of the blood-brain barrier and neurotropic character of the DSS isolate has any correlation to the disruption of the endothelium observed in humans suffering from vascular leakage. The three remaining amino acid substitutions unique for the DSS isolate were located in the NS protein 3 (S→P118), and 5 (T→I49, and S→N830). These differences could potentially alter the enzymatic activity of these proteins.

The DSS isolate seemed to persist longer *in vivo *since viral RNA was detected in a majority of mice on day 15 p.i. compared to the DF and DHF infected mice, and thereby exhibited infection kinetics differing from the two other DENV-1 isolates (DF and DHF) where viral RNA decreased after day 6 p.i. Thus, the DSS isolate could be regarded as more virulent than the two other DENV-1 strains, since a majority of the DSS-inoculated mice had detectable levels of viral RNA, infection was also less affected by the dose of inoculums and persisted longer in a majority of mice (Figure 1). The wild-type DENVs analyzed here could provide evidence for differences in virus replication, which in turn could influence the clinical outcomes of the infection, and eventually partitally explain some differences in virulence observed in humans.

These results confirm the *in vitro *findings of the same DENV-1 isolates, that showed that the strains derived from a DSS patient can be distinguished based on phenotypic characteristics from the isolates derived from a DF and DHF case [1]. We have previously shown that the DSS virus isolate exhibit much slower replication kinetics in mammalian Vero cells and an apoptotic response in mosquito cells, and we observed in this study that the DSS virus isolate persist longer *in vivo *with extensive neuroinvasion.

Cytokines involved in inflammation were quantified in serum collected from inoculated mice and compared in regard to DENV-1 isolate, virus titer inoculated, and time-point p.i. Overall, the levels of the proinflammatory cytokines (IFNγ, IL-1β, IL-2, IL-6, IL-10, IL-13, MCP-1, RANTES, and TNFα) quantified were low, which is most probably due to fact that BALB/c mice is poorly susceptible to wild-type DENVs. The mice did not develop clinical apparent disease, and showed only discrete lesions in internal organs. Common for several cytokines, however, was a dose-dependent response to inoculated virus (IFNγ, IL-6, IL-13, and RANTES). The highest levels of measured cytokines were also in general at the beginning of the experimental period (IFNγ, IL-10, and MCP-1 peaked at day 3 p.i.). A curious finding was that DF-inoculated mice had higher detectable serum levels of IL-10, MCP-1, and RANTES, which have been seen to be increased in patients with severe DHF and DSS [36-42]. The preference for the brain of the DHF-, and most notably the DSS-infected mice, could imply locally high levels of proinflammatory cytokines that do not circulate systemically. This could explain why the IL-10, MCP-1, and RANTES levels dominated in the DF-inoculated mice, since the thoracic and abdominal organs were the primary target for DF isolate. Cytokines like IL-1β, IL-2, and TNFα did not show any differences compared to mock-infected controls and this could be due to the transient nature of many cytokines as well as the limited susceptibility of DENVs in mice.

## Conclusions

We have previously shown *in vitro *that the isolate from a DSS case could be distinguished based on replication kinetics and apoptosis from isolates originating in milder cases. In this study we have extended the characterization of those clinical DENV-1 isolates derived from patients exhibiting the various forms of dengue illness. Despite their close relatedness, the DENV-1 isolate from a DSS case was phenotypically different from the other two isolates by significant brain invasiveness and a higher infectivity in BALB/c mice. The isolates from a DF and DHF case infected primarily lungs and liver, and to a limited extent brain and infection declined faster after day 6 p.i than in DSS-infected mice.

This is the first time different clinical South-East Asian DENV isolates have been directly compared for the *in vivo *characteristics in BALB/c mice. To define key elements involved in the virulence of these characterized DENV-1 phenotypes reverse genetics systems are needed. Additional low-passage strains of the different DENV serotypes of each clinical forms of dengue are required to fully decipher the complex mechanisms governing DENV pathogenesis.

## Materials and methods

### Virus

Three dengue serotype-1 (DENV-1) virus strains isolated from patient sera during a DENV outbreak in the Kampong Cham province, Eastern Cambodia, in 2007, were used. The isolates were obtained from patients experiencing the three distinct clinical forms of dengue disease: DF, DHF and DSS, according to the WHO classification [43]. Patient information regarding the analysed clinical DENV isolates is presented in previous work by Tuiskunen, A. et al., 2011. Blood samples were collected between day 2 and 6 after onset of disease and the serotypes were determined at the Institute Pasteur in Cambodia (IPC) by nested reverse transcriptase-polymerase chain reaction, according to Lanciotti procedure modified by Reynes et al. [44]. The virus isolates were obtained during the DENFRAME study, which has been approved by the Cambodian National Ethics Committee and patient's enrolment was subject to obtaining a written consent signed by the patients, or the under 16 year old patient's legal representatives.

Each DENV was isolated in *A. albopictus *mosquito cell line C6/36 (CRL 1660, ATCC), and thereafter propagated by one passage. The C6/36 cells were maintained in Leibovitz-15 medium, supplemented with 2% tryptose phosphate broth, and 5% fetal calf serum (Invitrogen, Stockholm, Sweden) and maintained at 28°C for 6 days. Cell culture supernatant was centrifuged for 10 min at 15000 rpm, titrated as previously described [1] and stored at -80°C in a solution of sucrose [1.5 M] and hepes [1 M]. Virus had not undergone any previous passage in mouse.

### Animals

The animal experimental procedures were approved by the Committee for Laboratory Animal Science of the Swedish Board of Agriculture (ethical permit ID no: 339/07).

Female BALB/c mice (Nova, Sollentuna, Sweden) age 6 weeks weighing 20 g were maintained on a standard laboratory diet with water *ad libitum *and housed under specific pathogen-free conditions at the animal facility of the Swedish Institute for Communicable Disease Control (SMI) in Stockholm, Sweden. Mice subjected to the same treatment were kept in groups of 4-5 mice per cage and handled according to the international guidelines for experimentation on animals. DENV-1 infected mice were housed in a biological safety level (BSL) 3 isolator, and negative control mice were housed in a BSL 2 facility.

### DENV-1 infection in mice

There were 99 mice in total divided into four groups with 27 mice each according to the 3 DENV-1 isolates and 18 mock infected negative controls (NC). Each group of 27 mice were subdivided into three groups of nine mice that were injected intravenously (i.v.) in the tail vein with a single dose (1.5 × 10^4 ^PFU/mL; 1.5 × 10^5 ^PFU/mL; or 1.5 × 10^6 ^PFU/mL, respectively) of infective DENV-1 without adjuvant. Similarly, the NC group of 18 mice received 150 uL of Leibovitz-15 medium supplemented with sucrose [1.5 M] and hepes [1 M] (table 4). The mice were monitored daily for clinical signs.

**Table 4:** Schematic overview of the experimental design.

DENV-1 isolate	DF			DHF			DSS			NC
Titer PFU/mL	**1.5 × 10**^**4**^	**1.5 × 10**^**5**^	**1.5 × 10**^**6**^	**1.5 × 10**^**4**^	**1.5 × 10**^**5**^	**1.5 × 10**^**6**^	**1.5 × 10**^**4**^	**1.5 × 10**^**5**^	**1.5 × 10**^**6**^	N.A
Day 3 p.i	3	3	3	3	3	3	3	3	3	9
Day 6 p.i	3	3	3	3	3	3	3	3	3	9
Day 15 p.i	3	3	3	3	3	3	3	3	3	9
Total no. of mice	27			27			27			18

Mice were divided in groups of three depending on DENV-1 isolate for inoculation; titer of inoculum; and time of euthanasia. There were in total 99 mice included in the study, whereof 81 were inoculated with infectious DENV-1.

N.A. = not applicable

Blood that was obtained via a cardiac puncture of mice immediately after euthanasia by 4.5 - 4.8% isoflurane gas inhalation on day 3; 6, and 15 p.i. All mice were immediately splenectomised after euthanasia and thereafter stored at -80°C.

### Detection of DENV RNA in BALB/c mice

To assess viral burden in tissues of infected mice, organs such as liver, heart, spleen, lungs, brain, and kidneys were harvested, weighed, and homogenized using Stainless Steel Beads (5.0 mm diameter) with a TissueLyser apparatus (Qiagen, Hilden, Germany). Total RNA from tissues was extracted immediately after dissection using the RNeasy Mini kit according to the manufacturer's instructions (Qiagen).

Mouse sera were obtained by centrifugation of whole blood for 3 minutes at 2000 g in microtainer EDTA tubes (Becton, Dickinson and Company, Temse, Belgium). Viral RNA from thawed aliquots of serum (140 uL) was extracted using QIAamp Viral RNA Mini kit (Qiagen). RNA samples, eluted in RNAse-free water, were stored at -80°C. Quantitative measurements of total viral RNA were performed as previously described [45].

### IFA for virus-specific antibody detection

An in-house immunofluorescence assay (IFA) was used as previously described [46] essentially to verify that all DENV-1 infected mice had raised a DENV specific antibody response.

### Cytokine dosage

Cytokine levels in mouse sera collected from all inoculated mice, including mock infected, were measured as duplicates with Milliplex Mouse Cytokine/Chemokine Panel (Millipore) in a Luminex 100 (Luminex, Bio-Rad, Sweden). The following cytokines and chemokines were analysed according to the manufacturer's instructions: IFNγ, IL-1β, IL-2, IL-6, IL-10, IL-13, MCP-1, RANTES, and TNFα.

### Statistical Analysis

To test the effect of virus, titer and days p.i. on the risk of DENV infection in mouse organs binomial regression with a log link was used. Parametric bootstrap with 10.000 replicates was drawn from an assumed binomial probability model. The effect of virus, titer and day between smaller and larger models were then evaluated by comparing the deviance, extracted from the replicates. The largest model, within each analysis of the infection risk in an organ, included the main effects virus, titer and day plus the interactions virus by titer, virus by day and titer by day.

Median regression was used to evaluate the relationship between virus isolate, day, and titer on the different cytokines. As most cytokines displayed highly right skewed distributions in some virus isolates the use of median estimates yielded more robust results than with the use of mean based methods. Day and titer were analyzed separately with two main models per cytokine: Cytokine = group + day + group by day interaction; and Cytokine = group + titer + group by titer interaction. P-values < 0.05 were used as statistically significant results. All analyses and graphs were performed using the statistical software R (version 2.13.1, 2011).

## Competing interests

The authors declare that they have no competing interests.

## Authors' contributions

Conceived and designed the experiments: AT, ILG. Performed the experiments: AT, MW. Analyzed the data: AT, ÅL, ILG. Wrote the paper: AT. Reviewed the paper: PB, ILG, ÅL. All authors read and approved the final manuscript.
